# Biologic‐Induced Paradoxical Psoriatic Alopecia: A Systematic Review

**DOI:** 10.1111/ajd.14600

**Published:** 2025-09-12

**Authors:** Yaron Gu, Deshan F. Sebaratnam, Mani Makhija, Joshua Farrell

**Affiliations:** ^1^ Faculty of Medicine and Health The University of New South Wales Kensington New South Wales Australia; ^2^ Department of Dermatology Liverpool Hospital Liverpool New South Wales Australia; ^3^ Kossard Dermatopathologists Macquarie Park New South Wales Australia

**Keywords:** alopecia, biological products, psoriasis, tumour necrosis factor‐alpha

## Abstract

A systemic review was completed of primary research articles reporting patient outcomes in paradoxical psoriatic alopecia in association with biologic therapy. Our search strategy identified 96 patients from 45 studies in addition to our own case. Our review indicates a higher prevalence of paradoxical psoriatic alopecia in younger female patients, most commonly secondary to TNF‐alpha inhibitors. Unlike alopecia secondary to psoriasis vulgaris, a mixed inflammatory infiltrate featuring lymphocytes, plasma cells and eosinophils was observed. Remission was observed in most patients, including approximately a third of whom continued biologic therapy.

**Trial Registration:** PROSPERO registration number: CRD42023471174

## 1 Introduction

Biologic therapy has revolutionised the management of several chronic inflammatory diseases [1, 2, 3, 4, 5, 6], but despite a generally favourable safety profile, paradoxical reactions have been reported. Paradoxical reactions are the development or worsening of immune‐mediated disorders that usually respond to the therapy administered. They often appear within weeks or months of the introduction of the inciting agent, and include paradoxical psoriasis, psoriatic arthritis, inflammatory bowel disease (IBD), eczematous eruptions, alopecia areata, lichen planus and hidradenitis suppurativa amongst others [7]. The most common of these reactions is paradoxical psoriasis, which has been reported in up to 5% of patients receiving tumour necrosis factor (TNF)‐α inhibitor therapy for IBD, inflammatory arthritis and psoriasis [8]. Paradoxical psoriasis may manifest as chronic plaque, pustular, palmoplantar or guttate psoriasis, as well as psoriatic alopecia [9, 10, 11].

Psoriasis itself may cause alopecia, either through creating dystrophic hair more susceptible to removal via friction from topical treatment, or via a localised telogen effluvium [9]. Histological changes seen in this instance include psoriatic changes of the interfollicular epidermis, as well as a high proportion of telogen and catagen hairs [9]. Psoriasis is also associated with alopecia areata [9]. However, paradoxical psoriatic alopecia appears to differ from both of these conditions [9, 11, 12]. It is associated with decreased follicular density, sebaceous gland atrophy, psoriasiform epidermal change, and, unique to paradoxical psoriatic alopecia, a conspicuous inflammatory infiltrate including eosinophils and plasma cells [11, 12]. It also follows that it is in response to the introduction of a biologic medication. The clinical appearance of paradoxical psoriatic alopecia may take the form of erythematous plaques with overlying scale and a decrease in hair density. The pull test may be positive. Trichoscopy may reveal inter‐ and perifollicular scale, arborizing and glomerular vessels, follicular units of single hairs and exclamation hairs [13, 14]. We first present a case of paradoxical psoriatic alopecia, before conducting a review of the literature to better define the characteristics of this condition.

## 2 Case Report

**FIGURE 1:**
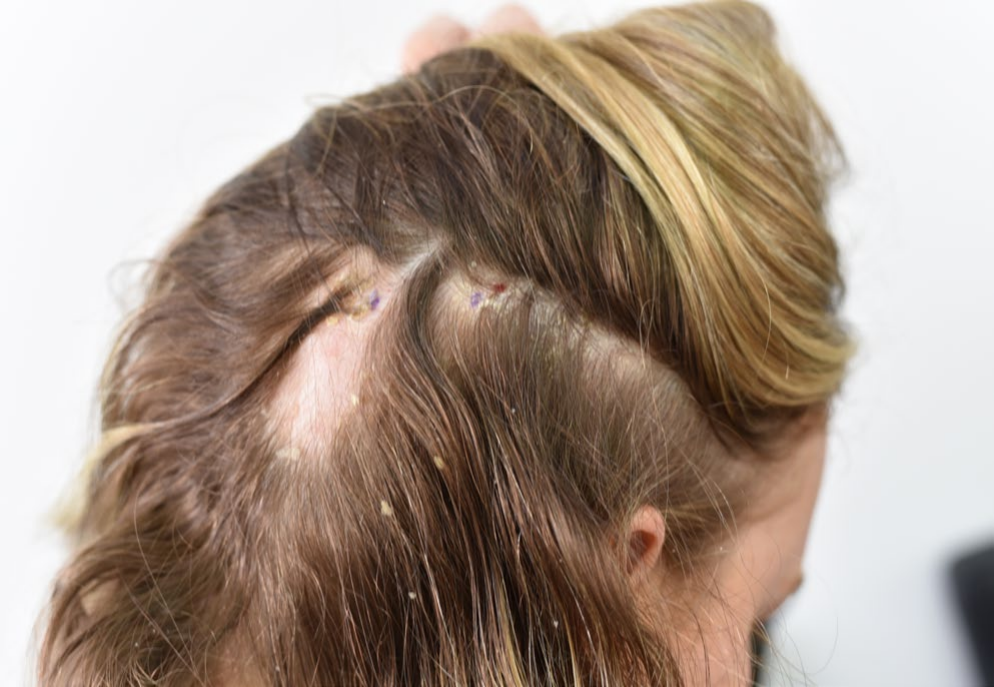
Well circumscribed patch of non‐scarring alopecia with focal pityriasis amiantacea.

**FIGURE 2:**
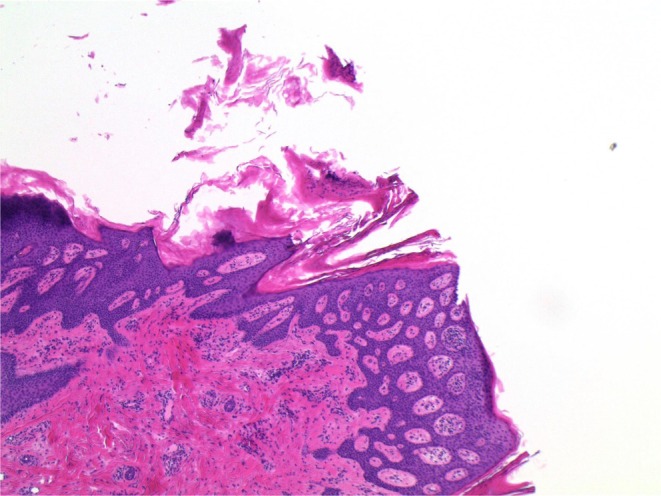
Parakeratotic mound with neutrophils in its summit (H&E, ×5).

**FIGURE 3:**
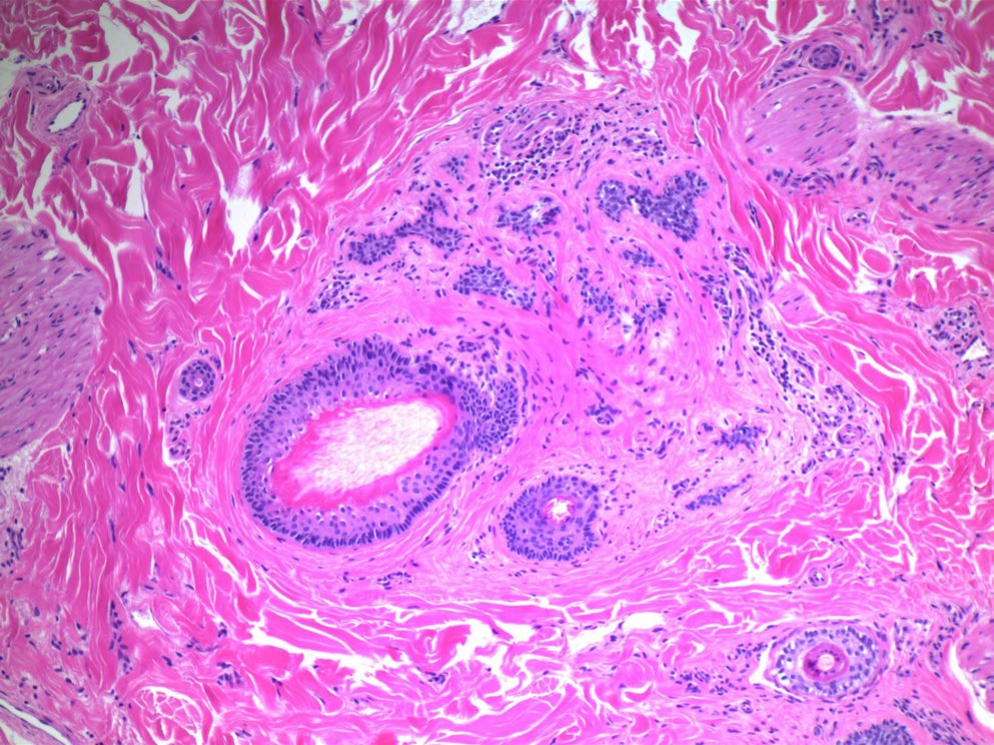
Miniaturised follicles and a catagen/telogen follicle with basaloid remnants of sebaceous glands (H&E, ×10).

We present a case of a 24‐year‐old female patient with a history of ankylosing spondylitis initially managed with adalimumab, who subsequently developed paradoxical psoriatic alopecia affecting her eyebrows and occiput. This resolved after cessation of adalimumab. Upadacitinib and certolizumab were then trialled without improvement in her ankylosing spondylitis. She was then commenced on ixekizumab, with the development of paradoxical psoriatic alopecia 4 weeks later (Figure 1). Scalp biopsy demonstrated parakeratosis with a neutrophilic infiltrate in keeping with a psoriasiform reaction (Figure 2), with the presence of sebaceous gland atrophy (Figure 3) and the presence of eosinophils in keeping with paradoxical psoriatic alopecia. With cessation of ixekizumab and topical clobetasol dipropionate shampoo three times per week, the alopecia remitted.

## 3 Review

While paradoxical psoriatic alopecia has largely been reported alongside TNF‐α inhibitors, there are increasing reports of this developing following exposure to more recently introduced biologics such as ustekinumab, secukinumab and, as in our case, ixekizumab. To date, no definitive treatment guidelines have been proposed and studies have been limited to the examination of biologics in a singular primary disease [15, 16]. Moreover, the demographic, clinical outcomes and histological features in patients with paradoxical psoriasis induced by non‐TNF‐α inhibitor biologics are limited [17], particularly in cases associated with alopecia [18]. This systematic review aims to synthesise the reported cases of biologic‐induced psoriatic alopecia to better define the clinical and histological features of this entity and to inform the management of these patients.

## 4 Methods

This systematic review was conducted in accordance with the protocol registered on the International Prospective Register of Systematic Reviews and is reported using the Preferred Reporting Items for Systematic Reviews and Meta‐Analysis guidelines.

### 4.1 Search Strategy

A literature search was conducted by two independent reviewers (Y.G. and J.F.) utilising electronic databases MEDLINE, EMBASE and PubMed. Databases were searched from inception to 10 October 2023 with language restricted to English. The search strategy employed combinations of the key terms: alopecia and biologics (Data S1).

### 4.2 Study Selection

Following deduplication, two reviewers (Y.G. and J.F.) screened the remaining records by title and for eligibility. Any discrepancies were resolved by a third reviewer (D.F.S.). Full‐text articles of remaining records were screened for eligibility. Primary research articles reporting patient outcomes following a diagnosis of paradoxical psoriatic alopecia in association with biologic therapy were included. The citations of retrieved studies were manually searched for additional articles.

### 4.3 Data Extraction

Data were extracted and tabulated from eligible articles including study characteristics, patient characteristics, personal and family history of psoriasis, causative biologic, time to onset, histological features and treatment outcomes.

## 5 Results

### 5.1 Literature Search

The search yielded 1216 records (Figure S1) with 981 records following the removal of duplicates and 122 records following initial screening. Ultimately 45 studies were included, comprising 27 case reports, 13 case series, 4 retrospective cohort studies and 1 prospective cohort study with a total of 96 patients.

### 5.2 Patient Characteristics

Including our patient, studies detailed biologic‐induced psoriatic alopecia in 97 patients from Asia, Europe, and North and South America. Patient characteristics are summarised in Table 1. Patients had a median age of onset of 26 years (range: 5–67 years). The male‐to‐female ratio was 20:62 (1:3.2) with a further 15 (15.4%) without specified sex. Of the 101 times biologics were used in these patients, TNFα inhibitors comprised a majority (*n* = 92; 91.1%) with interleukin (*n* = 8; 7.9%) and integrin inhibitors (*n* = 1; 1%) making up the remainder. Causative TNFα inhibitors included infliximab (*n* = 37; 40.2%), adalimumab (*n* = 34; 37%), certolizumab pegol (*n* = 5; 5.4%), etanercept (*n* = 1;1.1%) and the remainder were unspecified (*n* = 15; 16.3%). Of the eight instances where interleukin inhibitors were used, secukinumab, ixekizumab and ustekinumab were used twice (25%) while dupilumab and ustekinumab were each used once (12.5%). The most common indication for biologics was IBD (*n* = 63; 64.9%) of which 46 (73.0%) had Crohn's disease, 2 (3.2%) had ulcerative colitis, and 15 (23.8%) were unspecified. Other indications included psoriasis (*n* = 7; 7.2%), psoriatic arthritis (*n* = 5; 5.2%), juvenile idiopathic arthritis (*n* = 5; 5.2%), chronic recurrent multifocal osteomyelitis (*n* = 4; 4.1%), rheumatoid arthritis (*n* = 3; 3.1%), ankylosing spondylitis (*n* = 4; 4.1%) and Synovitis‐acne‐pustulosis‐hyperostosis‐osteitis syndrome (*n* = 2; 2.1%). Atopic dermatitis, hidradenitis suppurativa and sacroiliitis were the indications for one patient (1.0%) each.

**TABLE 1:** Study and patient characteristics.

	Number	Percentage
Total studies	45	
Case report	27	60
Case series	13	28.9
Retrospective cohort	4	8.9
Prospective cohort	1	2.2
Location		
Asia	10	22.2
Europe	20	44.4
North America	13	28.9
South America	2	4.4
Patient characteristics		
Total patients	97	
Age of onset (median, range)	26, 5–67	
Female	62	63.9
Male	20	20.6
Unknown	15	15.5
History of psoriasis		
Family history	3	3.1
Personal history	13	13.4
Biologic indication		
Inflammatory bowel disease	63	64.9
Crohn's disease	46/63	73
Ulcerative colitis	2/63	3.2
Unspecified	15/63	23.8
Ankylosing spondylitis	4	4.1
Atopic dermatitis	1	1
Chronic recurrent multifocal osteomyelitis	4	4.1
Hidradenitis suppurativa	1	1
Juvenile idiopathic arthritis	5	5.2
Psoriasis	7	7.2
Psoriatic arthritis	5	5.2
Rheumatoid arthritis	3	3.1
Sacroiliitis	1	1
SAPHO syndrome	2	2.1
Total biologics^a^	101	
TNFα inhibitor	92/101	91.1
Adalimumab	34/92	37
Certolizumab pegol	5/92	5.4
Etanercept	1/92	1.1
Infliximab	37/92	40.2
Unspecified TNFα inhibitor	15/92	16.3
Interleukin Inhibitor	8/101	7.9
Dupilumab (IL‐4/13)	1/8	12.5
Brodalumab (IL‐17A)	1/8	12.5
Ixekizumab (IL‐17A)	2/8	25
Secukinumab (IL‐17A)	2/8	25
Ustekinumab (IL‐12/23)	2/8	25
Integrin inhibitor	1/101	1
Vedolizumab	1/1	100
Concomitant systemic immunomodulator^b^	20	19.8
Azathioprine	7/20	35
Corticosteroid	6/20	30
Leflunomide	8/20	40
6‐Mercaptopurine	1/20	5
Methotrexate	8/20	40
Sulfasalazine	2/20	10

^a^ Number of biologics exceeds number of patients as some patients developed paradoxical psoriasis to a biologic and subsequently developed paradoxical psoriatic alopecia with a secondary biologic.

^b^ Total number of patients with immunomodulation is less than the number of immunomodulators used as some patients were on multiple therapies.

### 5.3 Paradoxical Psoriatic Alopecia Characteristics

The median duration of biologic therapy at the onset of disease was 7 months with a range from 3 weeks to 7 years. A personal history of psoriasis was reported in 13 (13.4%) patients and a family history in 3 (3.1%) (Table 2).

**TABLE 2:** Features of psoriasis, histology and management.

	Number	Percentage
Paradoxical psoriasis characteristics		
Duration of biologic therapy (median, range)	7 months, 3 weeks–7 years	
Personal history of psoriasis	13	13.4
Family history of psoriasis	3	3.1
Scalp biopsy performed	67	69.1
Sebaceous atrophy	15/67	22.4
Follicular atrophy	15/67	22.4
Neutrophils	23/67	34.3
Lymphocytes	51/67	76.1
Eosinophils	30/67	44.8
Plasma cells	19/67	28.4
Management		
Attempted biologic continuation	31	32.0
Success	9/31	29.0
Failure	22/31	71.0
Switching to another biologic	52	53.6
Biologic of choice for switching		
TNFα inhibitors	22	42.3
Adalimumab	11/22	50.0
Certolizumab pegol	3/22	13.6
Etanercept	3/22	13.6
Infliximab	5/22	22.7
Interleukin inhibitor	42	80.8
Canakinumab	1/42	2.4
Guselkumab	1/42	2.4
Ixekizumab	1/42	2.4
Secukinumab	2/42	4.8
Tocilizumab	2/42	4.8
Ustekinumab	29/42	69.0
Vedolizumab	2/42	4.8
Others	3	5.8
Abatacept	2/3	66.7
Upadacitinib	1/3	33.3
Cessation of biologic	35	36.1
Treatment outcomes		
Remission	87	89.7
Complete remission	35/87	40.2
Partial remission	52/87	59.8
No remission	10	10.3

#### 5.3.1 Histological Characteristics

Biopsies were performed and histological characteristics were described in 67 (69.1%) patients. Reported features included the presence of lymphocytes (*n* = 51; 76.1%), neutrophils (*n* = 23; 34.3%), eosinophils (*n* = 30; 44.8%), plasma cells (*n* = 19;28.4%), follicular atrophy (*n* = 15; 22.4%) and sebaceous atrophy (*n* = 15; 22.4%).

#### 5.3.2 Management and Treatment Outcomes

Immediate cessation of the causative biologic occurred in 36 (37.1%) patients while treatment was continued in 31 (32.0%) patients. While 9 (29.0%) patients successfully had remission of alopecia with continued biologic therapy, a majority (*n* = 22;71.0%) either did not achieve disease remission or had worsening of their disease. Amongst these 22 patients, 16 (72.7%) were ultimately switched to alternative biologics while the remainder ceased biologic therapy. Overall, an attempt to initiate an alternative biologic was trialled in 52 (53.6%) patients. Biologics of choice when switching to another included ustekinumab, adalimumab, etanercept, abatacept, certolizumab, infliximab, secukinumab, tocilizumab, vedolizumab, canakinumab, ixekizumab and guselkumab (Table 2).

Alopecia treatment included a range of topical therapies, systemic therapies and intralesional corticosteroids. Amongst the 97 patients, 87 (89.7%) achieved alopecia remission, of which 34 (39.1%) had complete remission and 52 (59.8%) had partial remission. Alopecia was permanent or continued to worsen in 10 (10.3%) patients.

## 6 Discussion

Our review demonstrates that paradoxical reactions occur in a range of conditions but are most commonly reported in the treatment of IBD. TNF‐α inhibitors account for almost all paradoxical psoriatic alopecia cases, with adalimumab and infliximab accounting for 70.3% of all cases. Although several interleukin inhibitors have been implicated, it is uncertain whether the smaller percentage of cases reflects their comparatively recent introduction with fewer patient years of exposure, or a lesser likelihood of precipitating psoriatic alopecia.

A female predominance was observed, which is consistent with prior literature on paradoxical reactions, although this may reflect the epidemiology of the primary inflammatory disease [15, 19]. Paradoxical psoriasis occurs most commonly between ages 31 and 55 [19] and around 70% within the first year of treatment [17]. However, as demonstrated in this review, there was significant variation in both age (5–67 years) and duration of biologic therapy (3 weeks to 7 years) at the onset of disease (Table S1).

Histological findings of paradoxical psoriatic alopecia have previously demonstrated resemblance to both idiopathic psoriatic alopecia and alopecia areata, prompting suggestions of potential overlap [20]. However, several histological distinctions have been suggested. The most common histological findings in TNF inhibitor‐induced psoriasis compared with psoriasis vulgaris have been reported as at least three dermal eosinophils per histologic section, neutrophils in the stratum corneum, neutrophils in the epidermis, papillary plate thinning and the absence of parakeratosis [11]. Other earlier studies emphasised characteristic features of plasma cells and eosinophils in the inflammatory infiltrate, which are rarely found in primary psoriatic alopecia [21]. Furthermore, the presence of CD4 and CD8^+^ lymphocytes may suggest a paradoxical reaction, thought to play a role in the follicular changes [22]. Additionally, psoriasiform epidermal changes and sebaceous gland atrophy assist in histological differentiation from biologic‐induced alopecia areata [23]. Sebaceous lobule atrophy has been proposed as a diagnostic feature of paradoxical psoriatic alopecia [12, 24] and 14 cases to date in addition to our case have reported this finding.

Paradoxical reactions may arise as de novo psoriasis, but flares or morphological changes in pre‐existing psoriasis can occur. Of the 97 cases of paradoxical psoriatic alopecia with data available, 13 (13.4%) reported a personal history of psoriasis and 12 of these patients experienced improvement in their alopecia. Amongst the 3 (3.1%) cases with data on family history available, all exhibited hair regrowth. This is consistent with previous reports on paradoxical psoriasis that often psoriasiform eruptions arising in patients with a history of psoriasis resolve on cessation of the offending agent [25].

The prognosis of paradoxical psoriatic eruptions is broadly favourable and many skin lesions partially or entirely resolve with topical therapy. Despite this, complete remissions are most commonly achieved by withdrawing the causative drug, with up to two‐thirds of cases ultimately resolving [26]. The paradoxical eruption is commonly a class effect, and so switching to another agent in the same class may either not provide improvement or may induce further psoriasis of the same subtype as the original paradoxical reaction. Although less common, paradoxical psoriatic alopecia may progress to cicatricial alopecia and thus require cessation of the causative agent [27].

Paradoxical psoriatic alopecia may also be dose‐related [28]. There is a report of one patient receiving adalimumab for Crohn's disease for 18 months before a dose increase to 40 mg weekly from 40 mg every second week was thought to have triggered the psoriatic alopecia [28].

The mechanism underlying paradoxical reactions remains to be elucidated. Previously suggested mechanisms include the modification of cytokine balance, activation of other inflammatory pathways, and a change in the polarisation of T‐cell response [26, 29]. For example, it has been theorised that inhibition of Th17 via IL‐17 may cause repolarization to Th2 cells, resulting in an eczematous paradoxical reaction [26]. They are also linked to a disequilibrium between TNF and type I interferon. They are characterised by a selective overexpression of type I interferon, dermal accumulation of plasmacytoid dendritic cells, and reduced T cell numbers [30]. Although it is unclear why some patients develop one manifestation rather than another, it has been suggested that the reason why only a small group of patients receiving TNF‐α inhibitors develop psoriasiform eruptions or alopecia might involve TNF receptor polymorphisms [23, 27, 31]. The involvement of other cytokines is suggested by previous reports that IL‐4/13 antagonises IL‐17A and IL‐17A mediated tight junctions, potentially explaining the psoriasiform features seen in the patients on dupilumab [32]. More recently, it has been suggested that IL‐33 may be responsible as it is present in both the epidermal keratinocytes as well as the keratinocytes in the outer root sheath of hair follicles. IL‐33 appears to upregulate epithelial cell proliferation and lymphocyte chemotaxis to cause the pathological changes seen in the hair follicles, and thus may present a therapeutic target [33].

This review is predominantly composed of Level IV and V evidence with an absence of clear patient selection methods. Additionally, histology was not obtained in all cases, and treatment outcomes and follow‐up were heterogeneous. Study follow‐up periods were varied and included time intervals from 1 month to 2 years, or were otherwise not stated. There was also heterogeneity in treatment approaches, with other systemic therapy used in 25 cases, topical therapy used in 41 cases, intralesional steroids in 4 cases and light therapy in 2 cases. Furthermore, for patients with resolution of psoriasis, underlying disease activity of inflammatory bowel or rheumatologic disease was not always reported. Additionally, patient outcomes were assessed subjectively, and this study likely underestimates the rate of complete remission as ambiguous cases were conservatively classified as partial remission.

## 7 Conclusion

Our review indicates a higher prevalence of paradoxical psoriatic alopecia in younger female patients, most commonly secondary to TNF‐alpha inhibitors. A mixed inflammatory infiltrate and sebaceous lobule atrophy were observed in contrast to primary psoriatic alopecia. Remission was observed in most patients, including approximately a third of whom continued biologic therapy.

## Conflicts of Interest

Deshan F. Sebaratnam has received consulting fees from Galderma, AbbVie, Amgen, Pfizer, Novartis, Janssen, Leo Pharma, Ego Pharmaceuticals, Viatris and Sun Pharma, and material support from Candela Medical and Heine Optotechnik. Deshan F. Sebaratnam is an Editorial Board member of the Australasian Journal of Dermatology and a co‐author of this article. To minimise bias, they were excluded from all editorial decision‐making related to the acceptance of this article for publication. Yaron Gu has received a scholarship from Ego Pharmaceuticals. Mani Makhija and Joshua Farrell have no conflicts to report.

## Supporting information


**Data S1:** ajd14600‐sup‐0001‐Supinfo.docx.

## Data Availability

The data that supports the findings of this study are available in the Supporting Information of this article.
