# Supplementary material for: Biologic‐Induced Paradoxical Psoriatic Alopecia: A Systematic Review
Source: Australas J Dermatol. 2025 Sep 12;66(8):441–7. doi: 10.1111/ajd.14600 (PMC12687687; doi:10.1111/ajd.14600)
Supplement: Supplementary file 1 — Data S1: ajd14600‐sup‐0001‐Supinfo.docx. [file AJD-66-441-s001.docx]

**Supporting Information**

**Contents**

Data S1. Search strategy for the systematic review

Figure S1. PRISMA Flow diagram

Table S1: Treatment outcomes on biologic therapy

**DATA S1: Search Strategy**

This systematic review was conducted in accordance with the protocol registered on International Prospective Register of Systematic Reviews (CRD42023471174) and is reported using the Preferred Reporting Items for Systematic Reviews and Meta-Analysis guidelines.

Electronic searches were performed using:

- Ovid MEDLINE
- Ovid EMBASE
- Pubmed

Databases were searched from inception to October 10, 2023. The search strategy employed permutations of the key terms: alopecia and biologics. Search terms utilised combinations of (“hair loss” OR “alopecia” or “bald*”) AND (“biologic*” OR [drug name]) AND (“psoria*”)

Drug names included:

- Biological products
- Biologic
- Monoclonal antibodies
- Infliximab
- Etanercept
- Adalimumab
- Golimumab
- Brodalumab
- Tumour Necrosis Factor
- Tumor Necrosis Factor
- TNF
- Certolizumab
- Ustekinumab
- Ixekizumab
- Secukinumab
- Risankizumab
- Tildrakizumab
- Dupilumab

Due to author linguistic proficiency, language was restricted to English. Following the selection process, the citations of included articles were assessed for further publications.

Records removed *before screening*:

Duplicate records removed (n = 235)

Records identified from:

MEDLINE (n = 118)

Embase (n = 974)

PubMed (n = 121)

Other sources (n = 3)

**Identification**

Records excluded

(n = 859)

Records screened

(n = 981)

Reports not retrieved

(n = 1)

Reports sought for retrieval

(n = 122)

**Screening**

Reports excluded:

Duplicate cases (n = 3)

Non-primary data (n = 16)

No psoriatic alopecia (n = 40)

No full text/not enough information (n = 16)

No biologic (n = 1)

Reports assessed for eligibility

(n = 121)

Studies included in review

(n = 45)

**Included**

**Figure S1:** PRISMA Flow diagram

| **Table S1** Treatment outcomes on biologic therapy | | | | | | | | | | |
| --- | --- | --- | --- | --- | --- | --- | --- | --- | --- | --- |
| References | Study type | Patient No. | Age | Sex | Biologic Indication | Biologic (concomitant immunomodulator) | Management | Response | | Outcome |
| Afanasiev et al. | Case series | 1 | 43 | F | Crohn's Disease | Adalimumab  (6-mercaptopurine) | Adalimumab discontinued. Initiation of 5-ASA for Crohn’s management. | PR | Near complete hair regrowth and residual scaling at 10-month follow-up. | |
|  |  | 2 | 60 | F | Rheumatoid Arthritis | Infliximab  (leflunomide) | Infliximab and leflunomide discontinued. Subsequent arthritis treatment with trials of cyclosporine, ustekinumab, apremilast, methotrexate and secukinumab. | CR | Alopecia resolved 10 months after cessation of infliximab and has remained clear at 2 years follow-up. | |
|  |  | 3 | 56 | F | Rheumatoid Arthritis | Adalimumab  (leflunomide) | Adalimumab discontinued. Initiated abatacept for rheumatoid arthritis management.  Clobetasol foam to treat psoriatic alopecia. | PR | Hair regrowth one month after cessation of adalimumab but her hair texture was curlier than before. | |
| Andrisani et al. | Case report | 1 | 26 | F | Crohn's Disease | Infliximab | Infliximab discontinued.  Initiation of topical clobetasol propionate and oral prednisone.  Initiation of ustekinumab. | CR | Progression of scalp alopecia with corticosteroid regimen.  Complete remission of scalp psoriasis at 6 month follow-up after commencement of ustekinumab. | |
| Aragon-Miguel et al. | Case report | 1 | 45 | F | Sacroiliitis | Certolizumab Pegol | Topical clobetasol for 8 weeks with no improvement, thus certolizumab pegol was discontinued.  Initiation of ustekinumab. | CR | Persistent alopecia with topical corticosteroid regimen.  Complete hair regrowth after 16 weeks of ustekinumab. | |
| Baniel et al. | Case series | 1 | 25 | F | Crohn's Disease | Adalimumab | Adalimumab discontinued.  Initiation of topical corticosteroids. | PR | Rapid, significant improvement after initiation of topical corticosteroids. | |
|  |  | 2 | 39 | F | Psoriatic Arthritis | Adalimumab | Initiation of topical corticosteroids.  Switching adalimumab for infliximab. | PR | Almost complete resolution with switch to infliximab. | |
|  |  | 3 | 64 | F | Psoriatic Arthritis | Adalimumab | 30 treatments of NBUVB with minimal improvement.  Adalimumab was subsequently switched with ustekinumab. | NR/LR | Switching from adalimumab to ustekinumab had no effect on the extent of alopecia | |
|  |  | 4 | 24 | M | Crohn's Disease | Infliximab | Continuation of infliximab with addition of topical calcipotriol-betamethasone and acitretin.  Subsequent transition to ustekinumab. | CR | Nil improvement with continuation of infliximab.  Complete remission following switch to ustekinumab. | |
|  |  | 5 | 22 | F | Crohn's Disease | Adalimumab | Discontinuation of adalimumab and addition of methotrexate and turban PUVA.  Initiation of ustekinumab. | CR | Worsening with adalimumab discontinuation and initiation of methotrexate and PUVA.  Complete resolution of skin findings following ustekinumab initiation. | |
|  |  | 6 | 22 | F | Crohn's Disease | Infliximab | Addition of topical treatment with coal tar, topical corticosteroids.  Addition of topical steroids. | CR | Slight improvement with topical treatments and complete resolution with addition of methotrexate. | |
| Bonomo et al. | Case report | 1 | 10 | F | Crohn's Disease | Infliximab | Initiation of fluocinonide 0.05% solution for scalp psoriasis.  Addition of oral methotrexate after two weeks.  After additional four weeks, the patient transitioned from infliximab to ustekinumab.  Initiation of NBUVB alongside ustekinumab. | CR | Minimal improvement with topicals.  Worsening with methotrexate.  Complete hair regrowth at one year follow-up. | |
| Campbell et al. | Case series | 1 | 5 | F | Chronic recurrent multifocal osteomyelitis | Infliximab (corticosteroid, methotrexate) | Infliximab discontinued.  Initiation of topical therapies for psoriasis.  Initiation of etanercept and canakinumab for CRMO with no improvement.  Infliximab was restarted and ustekinumab was added with improvement for one year but ustekinumab was later discontinued due to myalgias.  Sequential trials of adalimumab and tocilizumab resulted in worsening of psoriasis and CRMO, respectively.  Golimumab was initiated. | PR | Minimal response, worsening or intolerable adverse effects with adalimumab, tocilizumab, infliximab and ustekinumab.  At her most recent clinical visit, CRMO was in complete remission and only had mild alopecia with golimumab.  Topical corticosteroids were continued throughout. | |
|  |  | 2 | 12 | F | Chronic recurrent multifocal osteomyelitis | Infliximab (corticosteroid, methotrexate) | Infliximab dose was increased due to breakthrough CRMO pain, however, her psoriasis worsened.  Infliximab was discontinued and pamidronate and methotrexate were initiated. | CR | Psoriasis was completely resolved 5 months after infliximab discontinuation. | |
|  |  | 3 | 11 | F | Chronic recurrent multifocal osteomyelitis | Adalimumab (corticosteroid, methotrexate, leflunomide) | Initiation of topical corticosteroids and methotrexate with minimal improvement.  Adalimumab was then discontinued and pamidronate infusions were initiated. | PR | Almost complete resolution of psoriasis after 2 months and hair regrowth at 5 months after adalimumab discontinuation. | |
|  |  | 4 | 11 | F | Crohn's Disease | Infliximab (corticosteroid, methotrexate, sulfasalazine) | Topical corticosteroids were initiated with minimal improvement until discontinuation of infliximab.  Meloxicam and sulfasalazine initiated for CRMO with minimal improvement.  Pamidronate with adalimumab resulted in recurrence of paradoxical psoriasis.  Addition of topical corticosteroids and tar-based shampoo.  Adalimumab doses were administered at a lower frequency (from every 2 weeks to 3 weeks. | PR | Complete hair regrowth and resolution of scalp rash. Persistent palmar rash manageable with topical corticosteroids. | |
|  |  | 5 | 8 | F | Chronic recurrent multifocal osteomyelitis | Infliximab (corticosteroid, pamidronate, methotrexate) | Discontinuation of infliximab and Initiation of topical corticosteroids and tar-based shampoo. | PR | Psoriasis improved over the next month. Minimal plaques on the scalp and extremities, managed with topicals. | |
| Carrasquillo et al. | Case report | 1 | 12 | F | Crohn's Disease | Adalimumab (azathioprine)  + Ustekinumab | Initial treatment with adalimumab lead to paradoxical psoriasis.  Initiation of mometasone lotion for scalp lesions and triamcinolone cream for body lesions.  Switched to ustekinumab which improved skin lesions but lead to psoriatic alopecia.  Mometasone switched to clobetasol lotion and continuation with ustekinumab. | PR | Partial improvement of skin lesions with ustekinumab but worsening of scalp lesions leading to alopecia.  At 6-months follow-up, well controlled Crohn’s disease and considerable hair regrowth. | |
| Craddock et al. | Case report | 1 | 21 | F | Crohn's Disease | Adalimumab (azathioprine) | Continuation of adalimumab.  Intralesional triamcinolone acetonide monthly for three months. | CR | Progressive resolution with monthly corticosteroid injections. | |
| Doyle et al. | Case series | 1 | 21 | F | Crohn's Disease | Infliximab | Continuation of infliximab.  Initiation of intralesional triamcinolone injections and topical steroids. | PR | Significant improvement in alopecia with intralesional and topical corticosteroids.  Plaques elsewhere continued to flare. | |
|  |  | 2 | 27 | F | Crohn's Disease | Infliximab | Initiation of topical corticosteroids and NBUVB.  Switched from infliximab to certolizumab pegol. | PR | Alopecia resistant to NBUVB and topical corticosteroids but improvement with body lesions.  Hair regrowth following switching to certolizumab pegol. | |
|  |  | 3 | 39 | F | Crohn's Disease | Adalimumab | Initiation of topical clobetasol. | PR | Dramatic improvement in scaly plaques and alopecia. | |
| El Shabrawi-Caelen et al. | Case series | 1 | 19 | F | Crohn's Disease | Adalimumab | Withdrawal of adalimumab. | CR | Complete hair regrowth after adalimumab discontinuation. | |
|  |  | 2 | 31 | F | Crohn's Disease | Adalimumab | Histopathology noted a scarring alopecia.  Management is not described. | NR/LR | Scarring alopecia. | |
| Ferraresso et al. | Case report | 1 | 27 | F | Crohn's Disease | Certolizumab Pegol | Discontinuation of certolizumab pegol.  Initiation of topical clobetasol unguent and coal tar shampoo.  After several months, infliximab was initiated for her Crohn’s disease. | PR | Significant improvement and hair regrowth after 4 weeks of topical therapies.  Infliximab demonstrates good control of Crohn’s disease to date. | |
| Gawdzik et al. | Case report | 1 | 34 | F | Ankylosing Spondylitis | Certolizumab Pegol | Initiation of topical corticosteroid, natamycin and neomycin cream and salicylic oil.  Discontinuation of certolizumab pegol.  Patient was admitted and initiated with once daily IV hydrocortisone 200mg, oral antiobiotics, NSAIDs, topical boric acid solution and betamethasone with gentamycin cream.  Secukinumab was added – after two doses, new lesions developed.  Methotrexate was added with secukinumab. | PR | Nil improvement with topical therapies.  Continual exacerbation despite cessation of certolizumab pegol.  Only mild improvement with IV corticosteroids.  Psoriasis worsened with secukinumab alone.  With secukinumab and methotrexate, the patient demonstrated regrowth of hair after two months. | |
| Groth et al. | Case series | 2 | 14 | F | Juvenile idiopathic arthritis | Adalimumab | Patient was switched to etanercept and later to tocilizumab. | PR | No change following switch to etanercept.  Significant improvement after switch to tocilizumab. | |
|  |  | 6 | 13 | F | Juvenile idiopathic arthritis | Infliximab (methotrexate) | Discontinued infliximab. | CR | Complete resolution following discontinuation of infliximab. | |
|  |  | 7 | 11 | F | Juvenile idiopathic arthritis | Adalimumab | Switch to ustekinumab. | CR | Complete resolution following switch to ustekinumab. | |
|  |  | 9 | 8 | F | Juvenile idiopathic arthritis | Infliximab (methotrexate) | Switch to adalimumab. | PR | Partial improvement with switch to adalimumab. | |
| Guedes et al. | Case report | 1 | 34 | F | Crohn's Disease | Adalimumab + Vedolizumab (corticosteroids) | Extensive exudative scalp lesions developed with adalimumab which was subsequently discontinued. Topical steroids were initiated.  Methotrexate was continued with complete resolution of skin lesions and Crohn’s disease but was later stopped due to pregnancy planning.  Vedolizumab and systemic corticosteroids were initiated due to intestinal flare. Clinical remission was achieved after 10 months with vedolizumab monotherapy (after a tapering corticosteroid regime).  Two months later, there was reappearance of extensive scalp lesions and peri-fistula psoriatic lesions. Topical therapies were initiated and vedolizumab was discontinued. |  | Further worsening of exudative scalp lesions despite topical steroid therapy.  After the second infusion of vedolizumab, the patient only reported mild hair loss however had subsequent worsening.  While topical therapies were unsuccessful, improvement was seen after one month of vedolizumab cessation. | |
| Hosokawa et al. | Case report | 1 | 67 | M | Psoriasis | Brodalumab | The patient initially trialled adalimumab treatment but had secondary failure – thus, he was switched to brodalumab.  Following a paradoxical reaction to brodalumab, the patient was switched to guselkumab. | PR | Hair regrowth across the entire scalp and improvement of psoriatic erythema following switch to guselkumab. | |
| Ishii-Osai et al. | Case report | 1 | 60 | M | Psoriasis | Infliximab | Infliximab discontinued.  Topical steroids and maxacalcitol were initiated with minimal response.  Cyclosporine was initiated. | CR | Marked improvement with cyclosporine and complete regrowth of hair after 12 weeks. Cyclosporine was tapered to 1.5mg/kg/d and no recurrence to date. | |
| Jeong et al. | Case report | 1 | 38 | M | Ulcerative Colitis | Adalimumab | Adalimumab discontinued.  Mesalazine initiated. | PR | Two weeks after cessation of adalimumab, psoriatic lesions and degree of hair loss improved. | |
| Kabbani et al. | Case report | 1 | 52 | F | Crohn's Disease | Adalimumab | Switch to ustekinumab.  Addition of clobetasol propionate ointment.  Smoking counselling. | CR | Marked improvement at 1-month.  Complete resolution of lesions and full hair regrowth on the scalp at 6-months. | |
| Kawashima et al. | Case report | 1 | 22 | M | Crohn's Disease | Infliximab | Infliximab discontinued.  Topical steroids were initiated with improvement.  Flare of Crohn’s disease prompted initiation of adalimumab. | PR | Improvement seen upon cessation of infliximab and initiation of topical steroids.  At 6 months from adalimumab initiation, there have been no eruptions. | |
| Koumaki et al. | Case report | 1 | 48 | F | Crohn's Disease | Adalimumab | Adalimumab discontinued.  Topical clobetasol propionate initiated.  Ustekinumab initiated. | CR | Complete hair regrowth within 6 months of ustekinumab initiation. No psoriasis or alopecia at one year follow-up. | |
| Lauro et al. | Case report | 1 | 25 | F | Psoriasis | Certolizumab Pegol | Certolizumab pegol discontinued.  Pulsed oral dexamethasone and topical clobetasol lotion were initiated. | PR | After 8 weeks, desquamative lesions on the scalp had resolved and no new psoriatic lesions had appeared. | |
| Li et al. | Retrospective cohort | 1 | 58 | F | SAPHO syndrome | Etanercept | Withdrawal of TNF-α inhibitor.  Initiation of topical tacalcitol and *Tripterygium wilfordii* Hook F. | PR | Most lesions resolved within 2 to 4 months, whereas some lesions required 1-2 years to disappear. | |
|  |  | 3 | 36 | M | SAPHO syndrome | Infliximab | Withdrawal of TNF-α inhibitor.  Initiation of topical calcipotriol and *Tripterygium wilfordii* Hook F. | PR | Most lesions resolved within 2 to 4 months, whereas some lesions required 1-2 years to disappear. | |
| Manni et al. | Case report | 1 | 30 | F | Crohn's Disease | Infliximab | Topical corticosteroids, keratolytics and emollients were ineffective and thus infliximab therapy was discontinued.  Cyclosporine was initiated along with local emollients. | PR | At two-months, complete remission of skin lesions and improvement in alopecia.  At four-months, persistence of partially resolving alopecia.  At six-months after discontinuation of infliximab and one-month after completion of cyclosporine treatment, alopecia patches exhibited almost complete regrowth of hair. | |
| Medkour et al. | Case report | 1 | 32 | M | Crohn's Disease | Infliximab | Discontinuation of infliximab and initiation of topical therapy.  Crohn’s disease remained stable with azathioprine. | CR | Complete hair regrowth 4-weeks after withdrawal of infliximab and daily topical clobetasol propionate.  No recurrence at 1 year follow-up. | |
| Megna et al. | Case report | 1 | 56 | F | Psoriatic Arthritis | Adalimumab | Switch from adalimumab to certolizumab. | CR | Significant improvement of skin and alopecia at 3 months with psoriatic arthritis under control.  Complete skin clearance and complete hair regrowth at 6 months and maintained at 12 month follow-up. | |
| Mele-Ninot et al. | Case report | 1 | 50 | M | Psoriasis | Adalimumab | Initiation of topical clobetasol demonstrated progression of psoriasis and thus adalimumab was discontinued. | PR | Psoriatic patches significantly improved at 6 months. | |
| Mihaelescu et al. | Case report | 1 | 17 | F | Crohn's Disease | Ustekinumab | Initiation of topical and intralesional corticosteroids failed to elicit clinical response, thus ustekinumab was discontinued.  Initiation of short course of oral prednisone.  Subsequent transition to vedolizumab and azathioprine. | PR | Improvement of scalp disease and sustained Crohn’s disease remission with azathioprine and vedolizumab. | |
| Mori et al. | Retrospective cohort | 2 | 14 | F | Crohn's Disease | Infliximab | Infliximab discontinued.  Initiation cyclosporine and adalimumab. | NR/LR | Relapsed with cyclosporine and adalimumab. | |
|  |  | 3 | 57 | F | Psoriatic Arthritis | Adalimumab | Adalimumab discontinued.  Initiated ustekinumab. | NR/LR | Relapsed with ustekinumab. | |
|  |  | 4 | 45 | M | Psoriatic Arthritis | Adalimumab | Adalimumab discontinued.  Initiated ustekinumab. | CR | Resolved with ustekinumab. | |
|  |  | 8 | 66 | F | Rheumatoid Arthritis | Certolizumab Pegol | Certolizumab pegol discontinued.  Initiated cyclosporine. | CR | Resolved with cyclosporine. | |
| Murphy et al. | Case series | 1 | Unknown | M | Crohn's Disease | Infliximab | Initiation of potent topical steroids and methotrexate were ineffective.  Switching from infliximab to adalimumab lead to further deterioration. Biologics were discontinued.  Oral zinc and dapsone was initiated. | PR | Oral zinc and dapsone resulted in clear skin.  At 6-months, they were withdrawn with no skin flare. | |
|  |  | 2 | Unknown | M | Crohn's Disease | Infliximab (azathioprine) | Initiation of topical steroids with some improvement.  Switch to adalimumab lead to worsening and he was then initiated on a tapering course of oral corticosteroids.  Subcutaneous methotrexate was poorly tolerated.  Cyclosporine had a partial response.  Initiation of ustekinumab. | CR | Faecal calprotectin was elevated to 1168 μg/g 8 months off anti‐TNF‐α; however Crohn’s disease and cutaneous symptoms are in remission following initiation of ustekinumab. | |
| Olbjorn et al. | Case series | 1 | 15 | F | Crohn's Disease | Infliximab | Switch from infliximab to ustekinumab.  Infliximab later reintroduced due to poor Crohn’s disease control.  Continued combination therapy for 7 years before continuing with infliximab monotherapy. | PR | Ustekinumab improved hair loss and skin lesions but lead to flare of Crohn’s disease.  Developed rectovaginal fistula and abscess on infliximab monotherapy.  Still has some psoriatic lesions (managed with topicals) but no hair loss. | |
|  |  | 2 | 17 | F | Crohn's Disease | Infliximab | Switch from infliximab to ustekinumab.  Infliximab later reintroduced due to poor Crohn’s disease control.  Continued combination therapy for two years before trialling ustekinumab monotherapy again.  Later switched to adalimumab and methotrexate but had flares of Crohn’s disease and psoriasis.  Again, trialled infliximab and ustekinumab.  Switched to vedolizumab monotherapy. | PR | Ustekinumab improved hair loss and skin lesions but lead to flare of Crohn’s disease.  Latter trial of ustekinumab monotherapy resulted in a Crohn’s disease flare.  Adalimumab and methotrexate had poor control of Crohn’s disease and lead to psoriasis.  Developed psoriatic lesions with infliximab and ustekinumab after one year.  Developed several small bowel strictures on vedolizumab. | |
|  |  | 3 | 16 | M | Crohn's Disease | Infliximab | Initiated combination of infliximab and ustekinumab for two years and ustekinumab monotherapy thereafter. | CR | Crohn’s disease in remission without psoriasis. | |
| Osorio et al. | Case series | 1 | 26 | F | Crohn's Disease | Infliximab | Initiation of topical treatment including corticosteroids, vitamin D derivatives, coal tar, salicylic acid and urea for the scalp.  After 42 months of persistent flares of scalp lesions, infliximab was switched to adalimumab.  Methotrexate was later added due to lack of improvement.  After 6 months of adalimumab, the patient was switched back to adalimumab due to flares of Crohn’s disease. | NR/LR | Infliximab and methotrexate have been continued for 1 year. The patient continues to have scalp lesions with mild alopecic patches and flexural involvement.  In fear of worsening Crohn’s disease, the patient refuses to suspend infliximab treatment. | |
|  |  | 2 | 31 | F | Crohn's Disease | Infliximab | Initiation of topical treatment including corticosteroids, vitamin D derivatives, coal tar, salicylic acid and urea for the scalp with mild improvement.  Infliximab was suspended after three months of topical therapies with complete hair regrowth.  She was subsequently treated with methotrexate and later azathioprine to manage her Crohn’s disease. | CR | Complete regrowth upon suspension of infliximab.  Continues treatment with azathioprine for Crohn’s disease. | |
|  |  | 3 | 23 | F | Crohn's Disease | Adalimumab | Initiation of topical treatment including corticosteroids, vitamin D derivatives, coal tar, salicylic acid and urea for the scalp.  She continues adalimumab. | PR | The patient continues to have mild scalp lesions which is sufficiently managed with topical treatment without suspension of adalimumab. | |
|  |  | 4 | 30 | M | Crohn's Disease | Adalimumab | Initiation of topical treatment including corticosteroids, vitamin D derivatives, coal tar, salicylic acid and urea for the scalp.  Discontinuation of adalimumab.  Initiation of low-dose oral steroids and methotrexate.  Methotrexate was suspended due to pancytopenia. | CR | No signs of scalp or inverse psoriasiform eruption at three-months after addition of methotrexate.  After suspension, the patient was still clear at 1 month follow-up. | |
|  |  | 5 | 25 | F | Crohn's Disease | Adalimumab | Initiation of topical treatment including corticosteroids, vitamin D derivatives, coal tar, salicylic acid and urea for the scalp.  Oral methotrexate was initiated and after three months was replaced with intramuscular methotrexate with minimal improvement.  After one month, prednisolone was initiated and then cyclosporine. | PR | The patient is improving after 4-weeks of cyclosporine treatment but continues to have diffused scalp erythema and localised. hyperkeratotic crusts. | |
| Ozkur et al. | Case report | 1 | 37 | F | Psoriasis | Adalimumab | Adalimumab discontinued and methotrexate initiated with adjuvant topical agents. | CR | One month later, there was complete hair regrowth and remarkable improvement in skin. | |
| Papadavid et al. | Case series | 1 | 30 | F | Ankylosing Spondylitis | Adalimumab | Discontinuation of adalimumab.  Initiation of topical steroids.  Initiation of cyclosporine. | PR | No response to topical steroids and discontinuation of adalimumab.  Improved with cyclosporine. | |
| Perman et al. | Case series | 1 | 7 | F | Juvenile idiopathic arthritis | Adalimumab | Adalimumab discontinued.  Initiation of intraarticular steroid injections, oral corticosteroids, abatacept and methotrexate.  Also started on oral antibiotics, fluocinolone scalp oil, solution and ointment, calcipotriene solution, Burrow’s solution soaks, local debridement and cyclosporine. | NR/LR. | Intermittent exacerbations with focal persistent plaques and persistent scarring alopecia at 18 months follow-up. | |
|  |  | 2 | 11 | M | Crohn's Disease | Infliximab | Infliximab continued.  Initiated on oral antibiotics, fluocinolone scalp oil, ciclopirox shampoo, fluocinonide solution, triamcinolone ointment to the body and desonide ointment to the genitals. | CR | Clear using topical therapy at 11-months follow-up. | |
|  |  | 3 | 16 | M | Ulcerative Colitis | Infliximab | Infliximab continued but later switched to adalimumab.  Topical treatments include fluocinolone scalp oil, shampoo, clobetasol ointment for palmoplantar lesions and triamcinolone for body. | CR | Clear using topical therapy at one-month. | |
|  |  | 4 | 14 | F | Crohn's Disease | Infliximab | Infliximab discontinued.  Initiated methotrexate.  Topical therapies include fluocinolone in peanut oil, mometasone lotion, triamcinolone cream, fluticasone ointment, desonide lotion and calcipotriene cream. | PR | Nearly clear with therapy at four-months. | |
|  |  | 5 | 18 | F | Crohn's Disease | Infliximab | Infliximab discontinued after 5 months.  Initiated 6-mercaptopurine.  Topical therapies included mupirocin cream, triamcinolone ointment and calcipotriene cream. | NR/LR | No improvement at five-months. | |
| Puig et al. | Case report | 1 | 47 | F | Psoriatic Arthritis | Adalimumab | Topical corticosteroids, photochemotherapy, cyclosporine, acitretin and etanercept were ineffective or poorly tolerated.  Ustekinumab was initiated alongside acitretin but was later discontinued due to diffuse deffluvium. | CR | Marked improvement at one-month.  Complete resolution of skin lesions with acceptable therapeutic control of arthritis at 16-months follow-up. | |
| Ribeiro et al. | Case series | 1 | 28 | M | Crohn's Disease | Infliximab | Initiation of clobetasol gel, coal tar shampoo, intralesional corticosteroids and tacrolimus.  Infliximab was continued. | CR | After 3 months, complete hair regrowth and remission of cutaneous lesions. | |
|  |  | 2 | 14 | F | Crohn's Disease | Infliximab | Initiation of coal tar shampoo, LCD lotion and mometasone on body lesions.  Infliximab was continued. | CR | After 5 months, there was hair regrowth and remission of cutaneous lesions. | |
| Shimokata et al. | Case report | 1 | 34 | M | Crohn's Disease | Infliximab | Infliximab was switched to adalimumab. | PR | Dramatic improvement in erythematous plaques, alopecia and Crohn’s disease following switch to adalimumab. | |
| Tan et al. | Case report | 1 | 62 | F | Psoriasis | Ixekizumab | Discontinuation of ixekizumab.  Initiation of tapering regime of systemic steroids. | PR | Mild regrowth of affected scalp and decreased scaling at 2-months follow-up. | |
| Tillack et al. | Prospective cohort study | 2 | 26 | F | Crohn's Disease | Adalimumab | Topical therapies yielded no improvement.  Adalimumab was subsequently switched to ustekinumab. | CR | Rapid improvement of skin lesions and complete healing after three months of therapy. | |
|  |  | 3 | 22 | F | Crohn's Disease | Infliximab | Topical steroids and salicylates yielded no improvement.  Infliximab was subsequently switched to ustekinumab | CR | Rapid regression and complete healing after two months of therapy. | |
|  |  | 4 | 20 | M | Crohn's Disease | Infliximab | Topical steroids and salicylates as well as a switch to adalimumab and later certolizumab were unsuccessful.  The patient was treated with ustekinumab. | PR | Marked improvement with ustekinumab after two injections. | |
|  |  | 7 | 40 | F | Crohn's Disease | Infliximab | Topical steroids and salicylates yielded no improvement.  Infliximab subsequently switched to ustekinumab. | PR | Significant clinical improvement in skin lesions following ustekinumab therapy. | |
| Tirelli et al. | Case series | 2 | 25 | M | Ankylosing Spondylitis | Adalimumab + Secukinumab | Initial treatment with etanercept followed by adalimumab for three years prior to development of paradoxical pustular psoriasis.  Switch to secukinumab induced the onset of focal scalp alopecia.  Tofacitinib was initiated. | PR | Good clinical response to tofacitinib. | |
|  |  | 3 | 37 | F | Psoriasis | Secukinumab | Topical treatment with clobetasol was initiated and biologic treatment was continued but was later withdrawn due to relapse.  Secukinumab was discontinued and two months later adalimumab was initiated. | PR | While topical clobetasol lead to significant improvement, readministration of secukinumab led to relapse.  Withdrawal of secukinumab lead to almost complete clinical recovery after two months. | |
| Toda-Brito et al. | Case report | 1 | 24 | F | Crohn's Disease | Adalimumab | Adalimumab discontinued.  Initiation of topical clobetasol and coal tar shampoo. | CR | Clearance of scalp lesions and complete hair regrowth within two months. | |
| Udkoff et al. | Case report | 1 | 23 | F | Crohn's Disease | Infliximab | Infliximab discontinued and oral minocycline commenced with subsequent progression of alopecia.  Initiation of topical betamethasone lotion, mineral oil, coal tar, ketoconazole, salicylic acid shampoo and continuation of oral minocycline. | CR | At 4-months, no scalp scale and all hair had returned.  Minocycline and topical therapies were subsequently discontinued.  Ustekinumab was initiated to treat Crohn’s disease which continues to be well controlled and without paradoxical reactions. | |
| Vignoli et al. | Case report | 1 | 46 | F | Hidradenitis Suppurativa | Adalimumab | Adalimumab discontinued.  Topical steroids provided poor response and ixekizumab was initiated. | CR | Complete resolution of alopecia at week 16 of ixekizumab. Later treated with topical clobetasol.  At one-year of ixekizumab therapy, the patient has not had relapse of psoriasis or hidradenitis suppurativa. | |
| Yanai et al. | Retrospective cohort | 1 | Unknown | Unknown | IBD (unspecified) | TNF-α inhibitor (unspecified) | Continuation of TNF-α inhibitor ± scalp steroid injections ± methotrexate | PR | “Success” n=1 | |
|  |  | 2 | Unknown | Unknown | IBD (unspecified) | TNF-α inhibitor (unspecified) | Discontinuation of TNF-α inhibitor ± scalp steroid injections ± methotrexate | PR | “Success” n=4 | |
|  |  | 3 | Unknown | Unknown | IBD (unspecified) | TNF-α inhibitor (unspecified) |  |  |  |  |
|  |  | 4 | Unknown | Unknown | IBD (unspecified) | TNF-α inhibitor (unspecified) |  |  |  |  |
|  |  | 5 | Unknown | Unknown | IBD (unspecified) | TNF-α inhibitor (unspecified) |  |  |  |  |
|  |  | 6 | Unknown | Unknown | IBD (unspecified) | TNF-α inhibitor (unspecified) | Discontinuation of TNF-α inhibitor ± scalp steroid injections ± methotrexate | NR/LR | “Fail” n=1 | |
|  |  | 7 | Unknown | Unknown | IBD (unspecified) | TNF-α inhibitor (unspecified) | Discontinuation of TNF-α inhibitor and switched to ustekinumab | PR | “Success” n=7 | |
|  |  | 8 | Unknown | Unknown | IBD (unspecified) | TNF-α inhibitor (unspecified) |  |  |  |  |
|  |  | 9 | Unknown | Unknown | IBD (unspecified) | TNF-α inhibitor (unspecified) |  |  |  |  |
|  |  | 10 | Unknown | Unknown | IBD (unspecified) | TNF-α inhibitor (unspecified) |  |  |  |  |
|  |  | 11 | Unknown | Unknown | IBD (unspecified) | TNF-α inhibitor (unspecified) |  |  |  |  |
|  |  | 12 | Unknown | Unknown | IBD (unspecified) | TNF-α inhibitor (unspecified) |  |  |  |  |
|  |  | 13 | Unknown | Unknown | IBD (unspecified) | TNF-α inhibitor (unspecified) |  |  |  |  |
|  |  | 14 | Unknown | Unknown | IBD (unspecified) | TNF-α inhibitor (unspecified) | Discontinuation of TNF-α inhibitor and switched to ustekinumab | NR/LR | “Fail” n=2 | |
|  |  | 15 | Unknown | Unknown | IBD (unspecified) | TNF-α inhibitor (unspecified) |  |  |  |  |
| Zhu et al. | Retrospective cohort | 1 | 31 | M | Atopic Dermatitis | Dupilumab | Discontinuation of dupilumab.  Initiation of topical steroids. | PR | Partial improvement. | |

^1^ NR/LR = No/limited response; PR = partial remission; CR = complete remission.

**Abbreviation:** IBD, inflammatory bowel disease; TNF-α, tumour necrosis factor-α; IL, interleukin; SAPHO, synovitis, acne, pustulosis, hyperostosis, osteitis

Supplementary References

1. Andrisani G, Marzo M, Celleno L, et al. Development of psoriasis scalp with alopecia during treatment of Crohn's disease with infliximab and rapid response to both diseases to ustekinumab. Eur Rev Med Pharmacol Sci. 2013;17:2831-6.
2. Aragon-Miguel R, Calleja-Algarra A, Vico-Alonso C, et al. Psoriatic alopecia-like paradoxical reaction to certolizumab pegol. Int J Dermatol. 2019;58:e118-120.
3. Baniel A, Peled A, Samuelov L, et al. Scarring Alopecia in Tumor Necrosis Factor-alpha Antagonists-Induced Scalp Psoriasis. J Psoriasis Psoriatic Arthritis. 2023;8:90-95.
4. Bonomo L, de Moll EH, Li L, et al. Tumor necrosis factor inhibitor-induced psoriasis in a pediatric Crohn's disease patient successfully treated with ustekinumab. J Drugs Dermatol. 2020;19:328-31.
5. Campbell JA, Kodama SS, Gupta D, Zhao Y. Case series of psoriasis associated with tumor necrosis factor-alpha inhibitors in children with chronic recurrent multifocal osteomyelitis. JAAD Case Rep. 2018;4:767-71.
6. Craddock LN, Cooley DM, Endo JO, Longley BJ, Caldera F. TNF inhibitor induced alopecia: an unusual form of psoriasiform alopecia that breaks the Renbok mold. Dermatol Online J. 2017;23(3).
7. Doyle LA, Sperling LC, Baksh S, et al. Psoriatic alopecia/alopecia areata-like reactions secondary to anti-tumor necrosis factor-α therapy: a novel cause of noncicatricial alopecia. Am J Dermatopathol. 2011;33:161-6.
8. El Shabrawi-Caelen L, La Placa M, Vincenzi C, et al. Adalimumab-induced psoriasis of the scalp with diffuse alopecia: a severe potentially irreversible cutaneous side effect of TNF-alpha blockers. Inflamm Bowel Dis. 2010;16:182-3.
9. Ferraresso M, Garlatti M, Perez-Chada L, Martin M, Mazzuoccolo L. Certolizumab-Induced Paradoxical Psoriatic Alopecia. J Psoriasis Psoriatic Arthritis. 2020;5:86-92.
10. Gawdzik A, Ponikowska M, Jankowska-Konsur A, et al. Paradoxical Skin Reaction to Certolizumab, an Overlap of Pyoderma Gangrenosum and Psoriasis in a Young Woman Treated for Ankylosing Spondylitis: Case Report with Literature Review. Dermatol Ther. 2020;10:869-79.
11. Groth D, Perez M, Treat JR, et al. Tumor necrosis factor-α inhibitor-induced psoriasis in juvenile idiopathic arthritis patients. Pediatr Dermatol. 2019;36:613-7.
12. Pereira Guedes T, Pedroto I, Lago P. Vedolizumab-associated psoriasis: until where does gut selectivity go? Rev Esp Enferm Dig. 2020;112:580-1.
13. Hosokawa Y, Hamada T, Ashida H, Ikeda M. Effective treatment with guselkumab for psoriatic alopecia as paradoxical reaction. J Dermatol. 2019;46:e302-3.
14. Ishii-Osai Y, Yoneta A, Mizugaki N, Takahashi H, Yamashita T. Infliximab treatment-induced paradoxical psoriasiform reaction in patient with psoriasis vulgaris showing positive lymphocyte transportation test reaction. JAAD Case Rep. 2015;1:230-3.
15. Jeong KM, Seo JY, Kim A, et al. Tumor necrosis factor-alpha inhibitor-associated psoriatic alopecia in a patient with ulcerative colitis: A case report and review of the literature. Ann Dermatol. 2021;33:82-5.
16. Kabbani M, El Sayed F. Adalimumab-Induced Psoriasis with Severe Alopecia. Skinmed. 2022;20:136-8.
17. Kawashima K, Ishihara S, Yamamoto A, et al. Development of diffuse alopecia with psoriasis-like eruptions during administration of infliximab for Crohn's disease. Inflamm Bowel Dis. 2013;19:e33-4.
18. Koumaki D, Koumaki V, Katoulis A, et al. Adalimumab-induced scalp psoriasis with severe alopecia as a paradoxical effect in a patient with Crohn's disease successfully treated with ustekinumab. Dermatol Ther. 2020;33:e13791.
19. Lauro W, Picone V, Abategiovanni L, et al. A case of psoriatic alopecia secondary to certolizumab pegol: clinical and trichoscopic evaluation. Int J Dermatol. 2023;62:e70-72.
20. Li C, Wu X, Cao Y, et al. Paradoxical skin lesions induced by anti-TNF-alpha agents in SAPHO syndrome. Clin Rheumatol. 2019;38:53-61.
21. Manni E, Barachini P. Psoriasis induced by infliximab in a patient suffering from Crohn's disease. Int J Immunopathol Pharmacol. 2009;22:841-4.
22. Medkour F, Babai S, Chanteloup E, et al. Development of diffuse psoriasis with alopecia during treatment of Crohn's disease with infliximab. Gastroenterol Clin Biol. 2010;34:140-1.
23. Megna M, De Lucia M, Gallo L, et al. Psoriatic Alopecia and Paradoxical Psoriasis Induced by Adalimumab Successfully Treated with Certolizumab: Clinical, Trichoscopic, and in vivo Reflectance Confocal Microscopy Features. Skin Appendage Disord. 2023;9:207-10.
24. Mihailescu M, Cibull T, Joyce J. Development of drug-induced psoriasiform alopecia in a pediatric patient on ustekinumab. J Cutan Pathol. 2021;48:1523-25.
25. Mori M, Tobita R, Egusa C, et al. Clinical background of patients with psoriasiform skin lesions due to tumor necrosis factor antagonist administration at a single center. J Dermatol. 2021;48:1745-53.
26. Olbjorn C, Rove J, Jahnsen J. Combination of Biological Agents in Moderate to Severe Pediatric Inflammatory Bowel Disease: A Case Series and Review of the Literature. Pediatr Drugs. 2020;22:409-416.
27. Osório F, Magro F, Lisboa C, et al. Anti-TNF-alpha induced psoriasiform eruptions with severe scalp involvement and alopecia: report of five cases and review of the literature. Dermatol. 2012;225:163-7.
28. Özkur E, Altunay İ K, Leblebici C, Topkarcı Z, Erdem Y. Adalimumab-induced scalp psoriasis with severe alopecia. Dermatol Ther. 2019;32:e13033.
29. Papadavid E, Gazi S, Dalamaga M, Stavrianeas N, Ntelis V. Palmoplantar and scalp psoriasis occurring during anti-tumour necrosis factor-alpha therapy: a case series of four patients and guidelines for management. J Eur Acad Dermatol Venereol. 2008;22(3):380-2.
30. Perman MJ, Lovell DJ, Denson LA, Farrell MK, Lucky AW. Five cases of anti-tumor necrosis factor alpha-induced psoriasis presenting with severe scalp involvement in children. Pediatr Dermatol. 2012;29:454-9.
31. Puig L, Morales-Munera CE, Lopez-Ferrer A, Geli C. Ustekinumab treatment of TNF antagonist-induced paradoxical psoriasis flare in a patient with psoriatic arthritis: Case report and review. Dermatol. 2012;225:14-7.
32. Ribeiro LB, Rego JC, Estrada BD, et al. Alopecia secondary to anti-tumor necrosis factor-alpha therapy. An Bras Dermatol. 2015;90:232-5.
33. Shimokata M, Namiki T, Tokoro S, et al. Case of psoriasiform and pustular eruptions in addition to alopecia as a paradoxical reaction induced by infliximab. J Dermatol. 2018;45:e331-3.
34. Tan TL, Taglia L, Yazdan P. Drug-induced psoriasiform alopecia associated with interleukin-17 inhibitor therapy. J Cutan Pathol. 2021;48:771-4.
35. Tillack C, Ehmann LM, Friedrich M, et al. Anti-TNF antibody-induced psoriasiform skin lesions in patients with inflammatory bowel disease are characterised by interferon-γ-expressing Th1 cells and IL-17A/IL-22-expressing Th17 cells and respond to anti-IL-12/IL-23 antibody treatment. Gut. 2014;63:567-7.
36. Tirelli L, Alfaro A, Citera G, Echevarría C. Nonscarring alopecia secondary to secukinumab. Actas dermosifiliogr. 2022;113:1011-13.
37. Udkoff J, Cohen PR. Tumor necrosis factor-induced alopecia: alternative pathology and therapy. Dermatol Online J. 2017;23:13030.
38. Vignoli CA, Gargiulo L, Sanna F, Narcisi A, Costanzo A. Adalimumab-induced paradoxical pustular psoriasis and alopecia successfully treated with ixekizumab in a patient affected by hidradenitis suppurativa. J Dermatolog Treat. 2023;34(1):2256905.
39. Yanai H, Amir Barak H, Ollech JE, et al. Clinical approach to skin eruptions induced by anti-TNF agents among patients with inflammatory bowel diseases: insights from a multidisciplinary IBD-DERMA clinic. Therap Adv Gastroenterol. 2021;14.
